# Sonographic assessment of pediatric chest wall thickness and width of the intercostal space: correlation with anthropometric data and implications for needle decompression

**DOI:** 10.1186/s13089-021-00226-6

**Published:** 2021-05-10

**Authors:** Tom Terboven, Ivette Betka, Christel Weiss, Marcus Rudolph, Tim Viergutz, Georg Leonhard, Michael Schöler

**Affiliations:** 1 Department of Anaesthesiology and Intensive Care Medicine, University Medical Center Mannheim, Theodor-Kutzer-Ufer 1-3, 68167 Mannheim, Germany; 2 Department of Medical Statistics, University Medical Center Mannheim, Theodor-Kutzer-Ufer 1-3, 68167 Mannheim, Germany; 3 DRF Stiftung Luftrettung gemeinnützige AG, Filderstadt, Germany

**Keywords:** Tension pneumothorax, Chest wall thickness, Needle decompression, Risk of injury, Sonography, Pediatric

## Abstract

**Background:**

Emergent needle decompression in children is a rare event for emergency medicine and critical care providers. Hereby, risk of injury of intrathoracic structures is high and knowledge of age-specific values of chest wall thickness and width of the intercostal space (ICS) is crucial to avoid injuries. Investigation of the correlation of chest wall thickness and width of the intercostal space with age and body dimension like weight and height could provide guidance on depth of insertion and choice of the needle.

**Methods:**

We performed a prospective observational clinical trial in a pediatric surgery operating room that included a convenient sample of children aged 0–10 years undergoing elective surgery. Chest wall thickness and width of the intercostal space were measured with ultrasound at 2nd ICS midclavicular line (MCL) and 4th ICS anterior axillary line (AAL). Correlation of these measures with age, height, weight, BMI and Broselow color was calculated. Furthermore, intra-class correlation coefficient was calculated as a measure of reproducibility and the presence of vital structures (e.g., heart, thymus gland, large pulmonary vessels) at the possible insertion sites for needle decompression was investigated.

**Results:**

Of 410 potentially eligible patients, 300 were included in the study. Correlation of chest wall thickness was moderate with weight (2nd ICS MCL: *r* = 0.57; 4th ICS MCL: *r* = 0.64) and BMI (*r* = 0.44 and *r* = 0.6) and was lower with age (*r* = 0.38 for both intercostal spaces), height (*r* = 0.42 and *r* = 0.40) and Broselow color (*r* = 0.42 and *r* = 0.38). Correlation of width of the ICS with anthropometric data was generally stronger, with height showing the strongest, albeit not really strong, correlation (*r* = 0.71 and *r* = 0.62). Intra-class correlation was excellent with an ICC of 0.93. Vital structures were significantly more often present at 2nd ICS MCL then at 4th ICS AAL (14 vs. 2 patients; *p* = 0.0042).

**Conclusions:**

Correlation of chest wall thickness and width of the intercostal space with anthropometric data is at most moderate. Insertion depth and width of the intercostal space can therefore not be predicted accurately from anthropometric data. Ultrasound assessment of the thoracic wall appears to be a reliable technique and could therefore assist in reducing the risk of injury and increasing decompression success.

*Trial registration* German clinical trials register, DRKS00014973, Registered February 11th 2019, https://www.drks.de/drks_web/navigate.do?navigationId=trial.HTML&TRIAL_ID=DRKS00014973

**Supplementary Information:**

The online version contains supplementary material available at 10.1186/s13089-021-00226-6.

## Background

Decompression of tension pneumothorax in children is a potentially life-saving but rather infrequent event in pediatric emergency and critical care medicine. Due to lack of training and experience in mini-thoracostomy, a procedure that requires surgical dissection of tissues, a physician experienced in this technique will mostly be required. Needle decompression appears to be less invasive and easier to perform and therefore still plays a role in high urgency situations, especially when a pediatric surgeon is not immediately available or when wire-guided chest drains are placed. Whereas in adults failure of the procedure, due to chest wall thickness (CWT) exceeding the length of the catheter used for decompression, is a common problem, needle length usually is not the problem in children [1, 2]. The chest wall is thin and the pleural space can easily be reached with common needles. In contrast to adults, intrathoracic structures like the heart, large vessels or the thymus gland are in close proximity to the insertion sites and can therefore be injured during needle decompression [3–5]. Laceration of the intercostal vessels by the decompression needle is a complication that has been described several times in adults and is even much more likely in the tiny intercostal spaces (ICS) in children [6–8]. Therefore, risk of injury by needle decompression appears to be a concern in children. Age-specific values for CWT and width of the ICS have been investigated before and showed a large variability within age groups [2, 3]. Knowing the amount of correlation of these measures with anthropometric data like age, height, weight and BMI could assist in predicting required insertion depth of the needle and choosing the appropriate needle size in an individual child. This correlation has not been studied extensively [2, 9].

We therefore assessed CWT and width of the ICS in children using ultrasound and tested for correlation of these measurements with age, height and weight. We furthermore investigated intra-rater reliability and assessed the presence of vital structures within the ultrasound window at the commonly recommended insertion sites.

## Methods

**Fig. 1:**
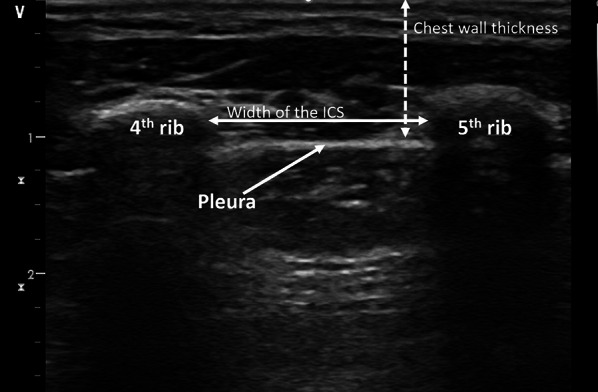
Ultrasound measurement of “chest wall thickness” and “width of the intercostal space”

This was a prospective study performed at a pediatric surgery operating room with a planned sample size of 300. All children aged 0–10 years presenting for elective surgery were eligible for inclusion and patients were recruited from February 15th 2019 to August 21st 2019 as a convenience sample without a fixed allocation to age groups. Parent’s written informed consent was obtained on the eve of surgery or prior to entering the operating room facilities. Patients with large intrathoracic pathologies, with an asymmetrical thorax or with previous intrathoracic surgery/thoracotomy or surgery of the chest wall were excluded. All measurements were performed and data collected immediately after induction of anesthesia and securing the airway. Chest wall thickness and width of the intercostal space were measured at the 2nd intercostal space at the midclavicular line (MCL) and at the 4th ICS at the anterior axillary line (AAL) on both sides of the thorax. The intercostal spaces were identified by palpation. When in doubt, identification of the correct site was confirmed via ultrasound. Width of the ICS was measured from the inferior border of the superior rib to the superior edge of the inferior rib (Fig. 1). The ultrasound probe was orientated cranio-caudally, perpendicular to the ribs. As a non-perpendicular orientation would lead to false-high values for width of the ICS, the probe was slightly rotated to find the minimal width of the ICS at the investigated insertion sites. Chest wall thickness was measured from skin surface to the pleural line at the superior edge of the inferior rib (Fig. 1). Compression of the subcutaneous tissue by force exerted via the ultrasound probe was recognized as a potential bias and was avoided as possible. All measurements were performed using the 12L linear probe (5–13 MHz) of a GE Healthcare (Solingen, Germany) Vivid S5 ultrasound machine. The investigations were performed by five investigators. All investigators are consultant anesthesiologists and licensed prehospital emergency medicine physicians experienced in ground-based and air rescue missions. They have successfully completed a basic emergency ultrasound course and have at least performed > 100 emergency ultrasound investigations of the chest prior to the start of the study. Moreover, they have received an additional 2-h hands-on training in sonography of the chest prior to the study by an instructor for emergency ultrasound who has performed > 5000 emergency ultrasound investigations and was one the investigators himself.

Children’s height and weight were extracted from the patients’ preoperative records as reported by the parents or measured at the ward. Body mass index (BMI) was calculated as weight (kilograms)/height^2^ (square meters). Additionally, the children were grouped into different categories based upon the Broselow length-based tape. Primary outcome was the evaluation of the correlation of CWT and width of the ICS with age, height, weight, BMI and Broselow category. Furthermore, the influence of each the mentioned variables on CWT and width of the ICS was tested using multivariable regression analysis and the presence of vital structures (thymus gland/heart/large pulmonary vessels) in the ultrasound window was assessed at the investigated insertion sites. Vital structures were only assessed as “seen” or “not seen” on the screen during evaluation of the chest wall. The distance to the pleura was not measured. As the human thorax, in the absence of pathologies, is symmetrical, we used the measured values at each side of the thorax to assess intra-rater reproducibility of the measured results.

Data were analyzed using SAS, release 9.4 (SAS Institute Inc., Cary, North Carolina, USA). Continuous variables are presented as mean ± standard deviation. Pearson’s correlation coefficient was used to test the correlation of CWT and width of the intercostal space with age, height, weight, BMI and Broselow category. The sample size of 300 enables to ensure a correlation between two quantitative variables assuming a Pearson’s correlation coefficient of 0.20 (with a power of 0.94). A binominal test was used to test for significance of differences of vital structures at the insertion sites. A test result with a p-value less than 0.05 was considered as statistically significant.

The study was registered at the German Clinical Trials Register (ID: DRKS00014973).

## Results

Of 410 patients that were potentially eligible, 110 were not included for various reasons (Additional file 1: Figure S1, Additional file 2: Table S1). The patients were examined by five different investigators with two investigators performing five investigations each, one performing 24 investigations, one performing 108 and one 158 investigations. Mean age of the study participants was 3.7 ± 3.0 years (range 0–10 years), 236 patients (78.7%) were male. Detailed demographic data and results of the ultrasound measurements of chest wall thickness and width of the ICS are shown in supplemental Additional file 3: Table S2 and Additional file 4: Table S3.

Correlation of chest wall thickness with the investigated body dimensions was moderate. The strongest correlation was found for weight of the children (2nd ICS *r* = 0.57, 4th ICS *r* = 0.64). BMI also showed a rather strong correlation at 4th ICS (*r* = 0.60) but not at 2nd ICS (*r* = 0.44). Age, height and Broselow categories showed a weaker relationship to chest wall thickness. Detailed results are shown in Table 1.

**Table 1:** Correlation of *“*Chest wall thickness*”* with anthropometric data (Pearson’s correlation coefficient; each *p* < 0.0001)

	2nd ICS MCL	4th ICS AAL
Age (years)	0.38	0.38
Weight	0.57	0.64
Height	0.42	0.40
BMI	0.44	0.60
Broselow	0.42	0.38

In multivariable regression analysis, weight was the variable which had the greatest influence on chest wall thickness at all insertion sites (partial *R*^2^ = 0.31–0.40). This influence was greater at the 4th ICS AAL (left: partial *R*^2^ = 0.4; right: partial *R*^2^ = 0.39) than at the 2nd ICS MCL (left and right: partial *R*^2^ = 0.31). The influence of height was also greater at 4th ICS AAL (left: partial *R*^2^ = 0.22; right: partial *R*^2^ = 0.25) than at 2nd ICS MCL (left: partial *R*^2^ = 0.08; right: partial *R*^2^ = not significant). Age, sex and Broselow category had statistically significant but very small influence. Detailed results are shown in Table 2.

**Table 2:** Multivariable regression analysis of factors influencing chest wall thickness (*p < 0.05)

Insertion site	Variable	Partial *R*^2^	Model *R*^2^
2nd ICS MCL _left_	Weight	0.31*	0.31*
	Height	0.08*	0.39*
	Broselow	0.02*	0.41*
	Sex	0.01*	0.42*
2nd ICS MCL _right_	Weight	0.31*	0.31*
	Age	0.10*	0.41*
	Sex	0.01*	0.42*
4th ICS AAL _left_	Weight	0.40*	0.40*
	Height	0.22*	0.62*
	Age	0.01*	0.63*
4th ICS AAL _right_	Weight	0.39*	0.39*
	Height	0.25*	0.64*
	Age	0.01*	0.66*

Regarding width of the intercostal space, the strongest correlation was found for height (2nd ICS MCL *r* = 0.71, 4th ICS AAL *r* = 0.62). Broselow color, age and weight showed similar correlations. No correlation was found with BMI. Detailed results are shown in Table 3.

**Table 3:** Correlation of *“*width of the intercostal space*”* with anthropometric data (Pearson`s correlation coefficient; each *p* < 0.0001 if not otherwise specified)

	2nd ICS MCL	4th ICS AAL
Age (years)	0.67	0.58
Weight	0.64	0.58
Height	0.71	0.62
BMI	0.03^†^	0.06_††_
Broselow	0.68	0.59

^†^*p* = 0.57; ^††^*p* = 0.29

In multivariable regression analysis for width of the ICS, height was the only variable that met the significance level for entry into the model at the four investigated insertion sites. Influence of height was stronger at 2nd ICS MCL (right: *R*^2^ = 0.5; left: *R*^2^ = 0.47) than at 4th ICS AAL (right: *R*^2^ = 0.37; left: *R*^2^ = 0.31) (Tab. 4).

**Table 4:** Multivariable regression analysis of factors influencing *“*width of the intercostal space*”* (*p < 0.05)

Insertion site	Variable	Partial *R*^2^	Model *R*^2^
2nd ICS MCL _left_	Height	0.47*	0.47*
2nd ICS MCL _right_	Height	0.50*	0.50*
4th ICS AAL _left_	Height	0.31*	0.31*
4th ICS AAL _right_	Height	0.37*	0.37*

## Intra-rater agreement

Overall intra-rater agreement was excellent with an ICC of 0.93 (CI 0.92–0.94). The lowest ICC for a single investigator was 0.75 (CI 0.47–0.89), the highest 0.99 (CI 0.97–0.99). The mean intra-rater difference for “Width of the ICS” was only 0.13 mm with a 95% confidence interval ranging from 0 to 0.34 at 2nd ICS MCL and 0.39 mm (CI 0.21–0.58) at 4th ICS AAL. For *“*chest wall thickness*”* mean intra-rater difference was 0.59 mm (CI 0.29–0.89) at 2nd ICS MCL and 0.04 mm (CI 0–0.24) at 4th ICS AAL.

## Vital structures

In 16 children, a vital structure was visible in the ultrasound window at one of the four investigated sites. Table 5 shows the distribution of the structures. Compared to 4th ICS AAL, vital structures were found significantly more often at the 2nd ICS MCL (*p* = 0.0042). A detailed description of patient characteristics and vital structures is shown in Table 6.

**Table 5:** Number of children with vital structures visible by ultrasound at the investigated sites

	Left	Right
2nd ICS MCL	12 (4.0%)	2 (0.7%)
4th ICS AAL	2 (0.7%)	0 (0%)

**Table 6:** Characteristics of patients with vital structures at insertion sites

Age	Weight (kg)	Vital structure	Insertion site
1 month	3.6	Thymus gland	2nd ICS MCL right
1 month	4.1	Heart	2nd ICS MCL left
3 months	6.1	Heart	2nd ICS MCL left
6 months	7.2	Thymus gland	2nd ICS MCL left
6 months	7.2	Thymus gland	2nd ICS MCL right
6 months	8.6	Heart	2nd ICS MCL left
12 months	11	Heart	4th ICS AAL left
12 months	11	Thymus gland	2nd ICS MCL left
1 year 3 months	11.7	Heart	2nd ICS MCL left
1 year 6 months	13.8	Heart	2nd ICS MCL left
1 year 7 months	12.7	Heart	2nd ICS MCL left
1 year 10 months	11.6	Heart	2nd ICS MCL left
3 years 5 months	11.8	Heart	2nd ICS MCL left
3 years 8 months	16	Heart	2nd ICS MCL left
3 years 8 months	16	Heart	2nd ICS MCL left
4 years 7 months	22	Heart	4th ICS AAL left

## Discussion

Decompression of tension pneumothorax in children is a rare but life-saving procedure that usually has to be performed under pressure. Due to the tiny spatial relationships, it carries a significant risk of injury to intrathoracic structures, especially in smaller children. Knowledge of chest wall thickness and width of the intercostal space enables the provider to choose the optimal decompression needle regarding length and bore but has only been investigated by few studies so far [2–4, 9]. Especially the correlation of age and body dimensions with these values has not been well investigated. Knowledge of these correlations could therefore assist in predicting the needed depth of insertion for decompression and minimize the risk of injury to intrathoracic structures and the intercostal vessels and nerve.

### Chest wall thickness and width of the intercostal space

In this study, we only found moderate correlations between measures of the chest wall relevant for needle decompression and body dimensions like age, weight and height. For chest wall thickness, the highest correlation was found with weight of the children (2nd ICS MCL *r* = 0.57, 4th ICS AAL *r* = 0.64). BMI also correlated well with CWT at 4th ICS AAL (*r* = 0.60) but not at 2nd ICS MCL (*r* = 0.44). Correlation for Broselow categories (2nd ICS MCL *r* = 0.42, 4th ICS AAL *r* = 0.38) and height (2nd ICS MCL *r* = 0.42, 4th ICS AAL *r* = 0.40) were lower and the lowest correlation was found for age (*r* = 0.38 at both insertion sites). Hossain et al. investigated CWT in a convenience sample of children presenting to the emergency department using ultrasound. Their results for correlation of CWT with weight (2nd ICS MCL: *r* = 0.55; 4th ICS AAL: *r* = 0.58) are comparable to ours. Correlation of CWT with age, height, BMI and Broselow color, however, was not investigated. Mandt et al. in a CT-based evaluation, also found the highest correlation between chest wall thickness and weight (2nd ICS-MCL: *r* = 0.53; 4th ICS-AAL: *r* = 0.45) and reported a lower correlation with height-based Broselow categories [2]. In most adult studies however, BMI, followed by weight, showed the strongest correlation with chest wall thickness [10–12]. In multivariable regression analysis in this present pediatric study, weight had the greatest influence on the children’s CWT, with a more pronounced influence at 4th ICS AAL. However, the effect size was low with a partial *R*^2^ ranging from 0.31 to 0.4. Both our own previous data as well as Mandt’s data showed a large variability of CWT within an age group [2, 3]. Given the at most moderate correlation of CWT with anthropometric data, the low effect size in multivariable regression analysis and the large variability within age groups, one seems to be able to roughly guess CWT from patient characteristics, but accurate prediction of the required insertion depth does not seem possible.

One of the many possible complications of needle thoracostomy that has previously been described in adults is laceration of the intercostal vessels [13]. The width of the intercostal space has, to the knowledge of the authors, not received any attention in adult studies and, even though injury appears much more likely in pediatric needle thoracostomy, was only investigated in one pediatric CT-based study [3]. The results of this study match very well with the results of the ultrasound measurements presented here. Previously recommended bore sizes for decompression in infants vary from 22 to 14G. The margin of safety the provider has when using a 14G needle in an infant is extremely small. One might even encounter an infant in which the use of a 14G needle unavoidably leads to laceration of the intercostal vessels. The mean diameter of the 4th ICS at AAL was 5.3 mm in this study, the outer diameter of a common 14G needle is 2 mm. This leaves a mean safety margin of 3.2 mm. Even a slight deviation from the correct angle of insertion in direction towards the upper rib might therefore lead to vessel injury. With an 18G or 22G needle the difference between the outer diameter of the needle and the width of the ICS is still small with 4 mm and 4.4 mm but increases by 25% or 37.5% compared to the 14G needle. Width of the ICS was moderately correlated with age, weight, height and Broselow color (*r* = 0.58–0.71). There was no correlation with BMI. For all variables, except BMI, correlation was higher at 2nd ICS then at 4th ICS. Multivariable regression analysis showed height being the only factor with significant influence on width of the ICS. This influence was more pronounced at 2nd ICS (*R*^2^ = 0.47–0.50) then at 4th ICS (*R*^2^ = 0.31–0.37). To our knowledge, there are no other studies investigating influences on width of the ICS in children or adults to which these findings could be compared.

### Reproducibility of the measurements

We found an excellent intra-rater agreement with an overall ICC of 0.93 (0.92–0.94). Mean intra-rater difference between left- and right-sided measurements was remarkably small (< 1 mm) at all insertion sites. To our best knowledge, no other studies examining intra-rater reliability of measurements of CWT or width of the ICS exist. Nanikawa et al. assessed intra-rater reliability of sonographic measurements of the thickness of the lateral abdominal wall muscles and found normal to excellent reliability of the measurements (ICC = 0.74–0.96) [14]. Keshwani et al. reported ICCs > 0.9 in measurements of inter-rectus distance in women with rectus diastasis [15]. These reports of high reliability of ultrasound measurements of the abdominal wall match well with our findings of good to excellent intra-rater agreement of sonographic assessment of CWT and width of the ICS.

### Vital structures

Vital structures were only detected in children younger than 5 years of age and significantly more often at the 2nd compared to the 4th ICS. The only structures identified by ultrasound were the heart and the thymus gland, large vessel were not detected. In children, different from adults, risk of injury to intrathoracic structures by the decompression needle seems to be a clearly bigger concern compared to insufficient needle length for decompression [1, 3, 16]. Nevertheless, failure of the procedure remains a problem. Quinn et al. report on 10 needle thoracocenteses performed prehospitally, of which 30% failed [17]. If this was due to inadequate insertion depth or other reasons like kinking of the catheters remains unclear. In their further course, all children required mini-thoracostomies for sufficient relieve of tension physiology. The authors therefore question the use of needle decompression in general and favor mini-thoracostomies [17, 18]. Up to the age of 10 years the intercostal width hardly exceeds 10 mm in the AAL [4]. Due to the small intercostal spaces, finger thoracostomy seems to be difficult in children, so a modified technique, e.g., with forceps, has to be used. It will however be hard to establish widespread sufficient training for this in children quite demanding procedure. Needle decompression therefore remains the technique of choice for many providers. The data from this study show that neither CWT nor width of the ICS can be accurately predicted from patient characteristics like age, weight or height. The rather high periprocedural risk of needle decompression in children in combination with the paucity of data has, in recent years, led to studies investigating this topic. As a result, current pediatric trauma guidelines recommend the 4th or 5th ICS AAL as the preferred insertion site together with the use of age appropriate decompression needles to reduce the risk of injury to vital structures [19]. Another possibility to reduce risk of injury and increase decompression success is using ultrasound. Extended Focused Abdominal Sonography for Trauma (eFAST) is an easy to learn and nowadays standard investigation in trauma care in all age groups and includes assessment of pleural sliding to rule out pneumothorax [20, 21]. Measurement of CWT and width of the ICS as well as recognition of vital structures close to the insertion site can easily be incorporated into ultrasound assessment of respiratory failure and preprocedural planning. One can then tailor the choice of needle size, especially diameter, and insertion depth to the individual patient and thereby increase decompression success and reduce risk of injury.

When ultrasound is not available, the provider has to keep in mind that age and height are rather poor predictors of CWT. The best, yet only moderate correlation was found for weight. As a rule of thumb, CWT only increases very little in the first 5 years of life and is slightly bigger at the anterior insertion site. Width of the intercostal space shows a more linear increase and is also bigger at 2nd ICS MCL.

## Limitations

There are several limitations to our study. It was a single-center study with a convenience sample of patients. This led to an uneven distribution of patients regarding age and sex. Furthermore, height and weight were extracted from the children’s medical records. We did not assess if these data were reported by the parents or measured by nurses. Because of this, they might differ from true values to a certain extent. In an emergency situation however, weight is usually reported by parents, guessed by the provider or extrapolated with the help of a pediatric tape and therefore will always deviate from true values to a certain degree. The study was conducted in an elective inpatient setting, not in emergency situations, and the measurements were performed after induction of anesthesia. Measuring the sometimes small distances might be more inaccurate in an awake and troubled or anxious child. This might have influence on the external validity of the presented data. Of note, the depth setting of the ultrasound probe was set at 3 cm as a starting point, but could be varied by the investigator. The excellent intra-rater agreement might have been influenced by the fact that the investigators were not blinded regarding the results of their initial measurements on the contralateral side. Inter-rater agreement was not assessed. Intrapleural pathology might distort anatomy and in case of tension pneumothorax move vital structures away from the insertion site. Lastly, there were two investigators that only performed 5 examinations each. These two investigators however are advanced and well trained in emergency sonography at all ages and their measurements were therefore included in this study.

## Conclusions

Weight had the greatest influence on chest wall thickness and height the greatest influence on width of the intercostal space. The correlation of chest wall thickness and width of the intercostal space with anthropometric data, however, was at most moderate and an accurate prediction of these measures therefore is hard. Sonography could prove to be helpful in choice of needle bore to minimize the risk of injury to the intercostal vessels and assess the required insertion depth to reach the pleural space for decompression.

## Supplementary Information

**Additional file 1: Figure S1.** Flowchart of patient recruitment and exclusion.

**Additional file 2: Table S1.** Reasons for exclusion.

**Additional file 3: Table S2.** Anthropometric data.

**Additional file 4: Table S3.** Chest wall thickness and width of the intercostal space (mm; mean ± standard deviation).

## Data Availability

All data generated or analyzed during the current study are available from the corresponding author on reasonable request.

## Abbreviations

ICS: Intercostal space
MCL: Midclavicular line
AAL: Anterior axillary line
CWT: Chest wall thickness
BMI: Body mass index
eFAST: Extended Focused Abdominal Sonography for Trauma

## References

[1] Laan D, Vu T, Thiels CA, Pandian TK, Schiller HJ, Murad MH, Aho JM (2016). Chest wall thickness and decompression failure: a systematic review and meta-analysis comparing anatomic locations in needle thoracostomy. Injury.

[2] Mandt MJ, Hayes K, Severyn F, Adelgais K (2019). Appropriate needle length for emergent pediatric needle thoracostomy utilizing computed tomography. Prehosp Emerg Care.

[3] Terboven T, Leonhard G, Wessel L, Viergutz T, Rudolph M, Schöler M, Weis M, Haubenreisser H (2019). Chest wall thickness and depth to vital structures in paediatric patients – implications for prehospital needle decompression of tension pneumothorax. Scand J Trauma Resusc Emerg Med.

[4] Leonhard G, Overhoff D, Wessel L, Viergutz T, Rudolph M, Schöler M, Haubenreisser H, Terboven T (2019). Determining optimal needle size for decompression of tension pneumothorax in children - a CT-based study. Scand J Trauma Resusc Emerg Med.

[5] Terboven T, Felcht J, Zahn K, Rudolph M, Schoeler M (2020). Verletzung der A. pulmonalis im Rahmen einer Nadeldekompression bei einem 5-jährigen Mädchen. Notfall und Rettungsmedizin.

[6] Kanai M, Sekiguchi H (2015). Avoiding vessel laceration in thoracentesis: a role of vascular ultrasound with color Doppler. Chest.

[7] Psallidas I, Helm EJ, Maskell NA, Yarmus L, Feller-Kopman DJ, Gleeson FV, Rahman NM (2015). Latrogenic injury to the intercostal artery: Aetiology, diagnosis and therapeutic intervention. Thorax.

[8] Mansour W, Samaha G, El Bitar S, Esper Z, Maroun R (2017). Intercostal artery laceration: rare complication of thoracentesis and role of ultrasound in early detection. Case Rep Pulmonol.

[9] Hossain R, Qadri U, Dembowski N, Garcia A, Chen L, Cicero MRA (2020). Sound and air: ultrasonographic measurements of pediatric chest wall thickness and implications for needle decompression of tension Pneumothorax. Pediatr Emerg Care.

[10] Hecker M, Hegenscheid K, Völzke H (2016). Needle decompression of tension pneumothorax: population-based epidemiologic approach to adequate needle length in healthy volunteers in Northeast Germany. J Trauma Acute Care Surg.

[11] Wax DB, Leibowitz AB (2007). Radiologic assessment of potential sites for needle decompression of a tension pneumothorax. Anesth Analg.

[12] Powers WF, Clancy TV, Adams A, West TC, Kotwall CA, Hope WW (2014). Proper catheter selection for needle thoracostomy: a height and weight-based criteria. Injury.

[13] Yacovone ML, Kartan R, Bautista M (2010). Intercostal artery laceration following thoracentesis. Respir Care.

[14] Nanikawa W, Miyazaki J (2019). Examination of the intrarater reliability of ultrasound measurements of the thickness of the lumbar and lateral abdominal muscles in the prone position. J Phys Ther Sci.

[15] Keshwani N, McLean L (2015). Ultrasound imaging in postpartum women with diastasis recti: intrarater between-session reliability. J Orthop Sports Phys Ther.

[16] Chang SJ, Ross SW, Kiefer DJ, Anderson WE, Rogers AT, Sing RF, Callaway DW (2014). Evaluation of 8.0-cm needle at the fourth anterior axillary line for needle chest decompression of tension pneumothorax. J Trauma Acute Care Surg..

[17] Quinn N, Palmer CS, Bernard S, Noonan M, Teague WJ (2019). Thoracostomy in children with severe trauma: an overview of the paediatric experience in Victoria, Australia. Emerg Med Australas.

[18] Teague W, Amarakone K, Quinn N (2019). Rule of 4’s: Safe and effective pleural decompression and chest drain insertion in severely injured children. Emerg Med Australas.

[19] Lehner M, Jung P, Olivieri M, Schmittenbecher PP (2021). Multiple trauma care in childhood — practical and pragmatic summary of the new guideline. Notfall und Rettungsmedizin.

[20] Vasquez DG, Berg GM, Srour SG, Ali K (2020). Lung ultrasound for detecting pneumothorax in injured children: preliminary experience at a community-based Level II pediatric trauma center. Pediatr Radiol.

[21] Brooke M, Walton J, Scutt D, Connolly J, Jarman B (2012). Acquisition and interpretation of focused diagnostic ultrasound images by ultrasound-naive advanced paramedics: Trialling a PHUS education programme. Emerg Med J.

