# Supplementary figures and images for: Sonographic assessment of pediatric chest wall thickness and width of the intercostal space: correlation with anthropometric data and implications for needle decompression
Source: Ultrasound J. 2021 May 10;13:25. doi: 10.1186/s13089-021-00226-6 (PMC8110626; doi:10.1186/s13089-021-00226-6)

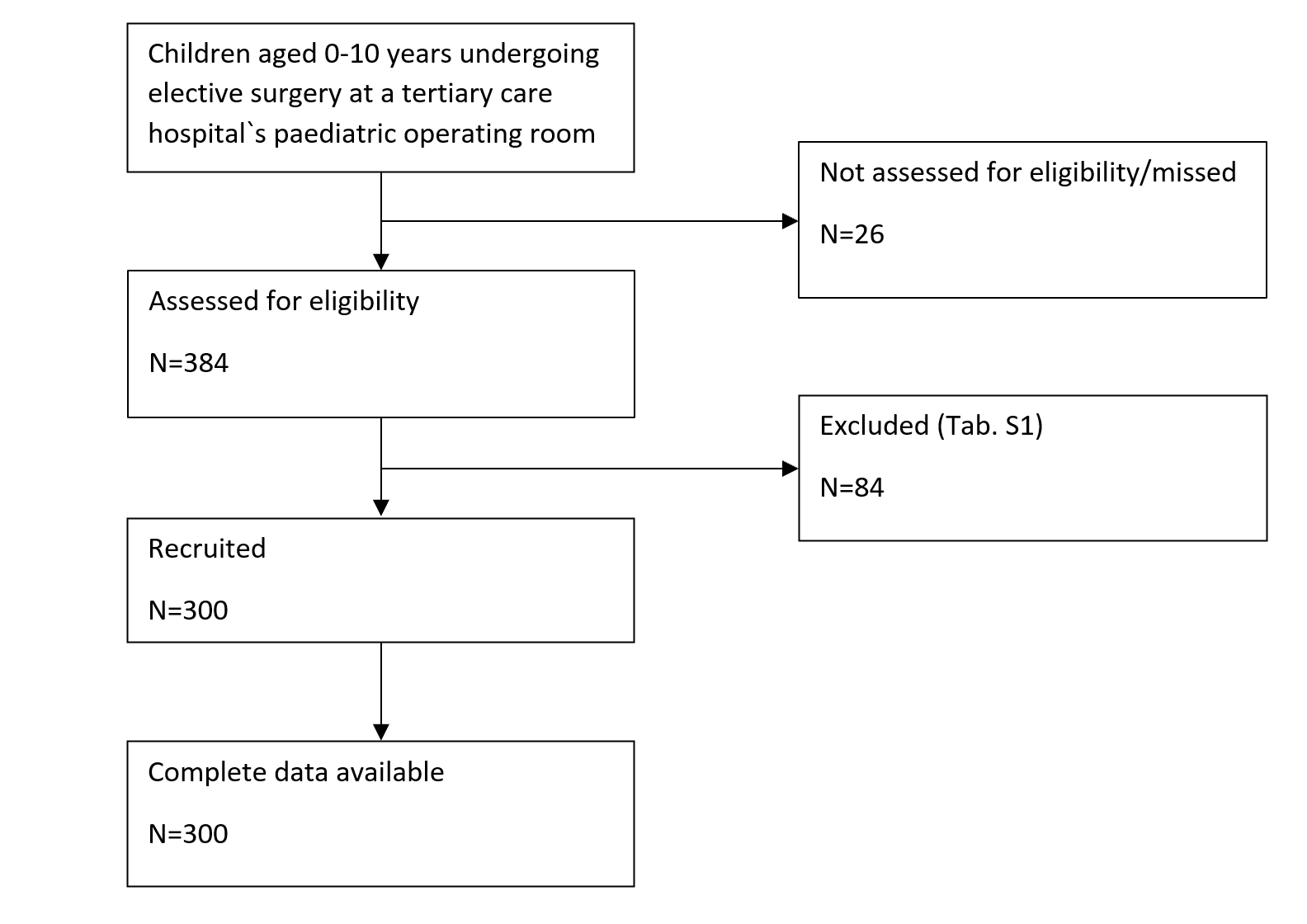

Supplement: Supplementary file 1 — Additional file 1: Figure S1. Flowchart of patient recruitment and exclusion. [file 13089_2021_226_MOESM1_ESM.png]
